# Supplementary material for: Numerical uncertainty in analytical pipelines lead to impactful variability in brain networks
Source: PLoS One. 2021 Nov 1;16(11):e0250755. doi: 10.1371/journal.pone.0250755 (PMC8559953; doi:10.1371/journal.pone.0250755)
Supplement: S1 File — (PDF) [file pone.0250755.s001.pdf]

S1 File

Supplemental Section S1 Graph Correlation. The following presents a quantification of deviations of generated connectomes from the reference execution, similar to shown in Figure 1. However, in this case, the “percent deviation” measure was replaced with the Pearson correlation coefficient. The correlations between observed graphs (Figure 4) across each grouping follow the same trend to as percent deviation, as shown in Figure 1. However, notably different from percent deviation, there is no significant difference in the correlations between dense or sparse instrumentations. By this measure, the probabilistic pipeline is more stable in all cross-MCA and cross-directions except for the combination of sparse perturbation and cross-MCA ($p < 0.0001$ for all; exploratory).

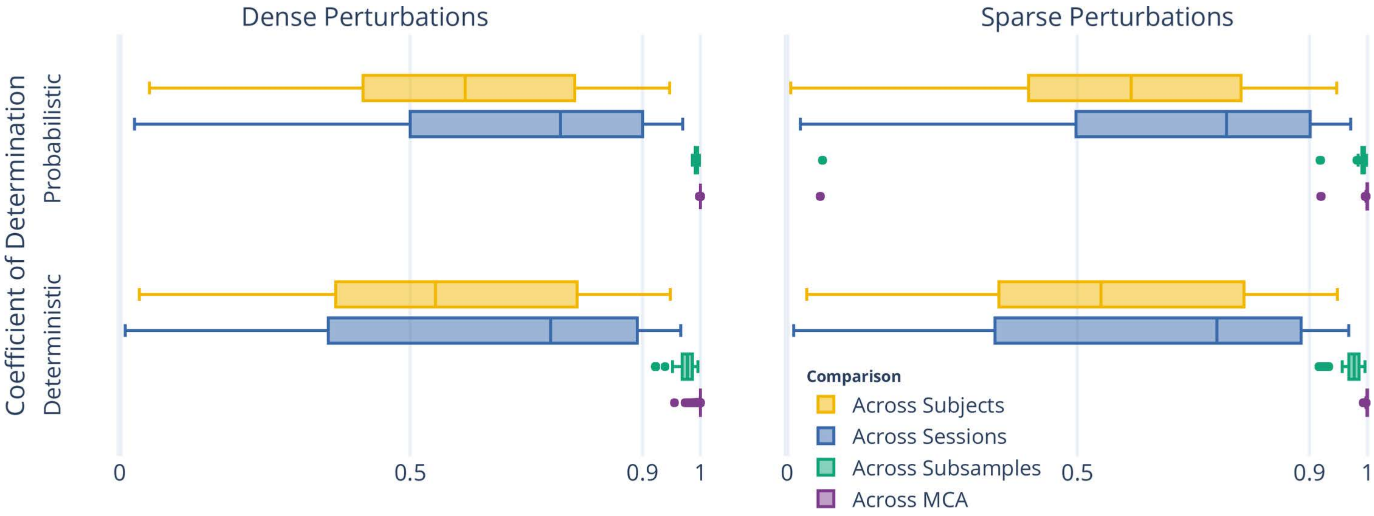

Fig 4. The correlation between perturbed connectomes and their reference.

The marked lack in drop-off of performance across these settings, inconsistent with the measures show in Figure 1 is likely due to the nature of the measure and the structure of graphs being compared. Given that structural graphs are sparse and contain considerable numbers of zero-weighted edges, the presence or absense of edges dominated the correlation measure where it was less impactful for the others. For this reason and others [1], correlation is not a commonly used measure in the context of structural connectivity, and thus this analysis was demoted to the supplement material.

Supplemental Section S2 Complete Discriminability Analysis

The complete discriminability analysis includes comparisons across more axes of variability than the condensed version. The reduction in the main body was such that only axes which would be relevant for a typical analysis were presented. Here, each of Hypothesis 1, testing the difference across subjects, and 2, testing the difference across sessions, were accompanied with additional comparisons to those shown in the main body.

Subject Variation Alongside experiment 1.1, that which mimicked a typical test-retest scenario, experiments 1.2 and 1.3 could be considered a test-retest with a handicap, given a single aqcuisition per individual was compared either across

Table 2. The complete results from the Discriminability analysis, with results reported as mean ± standard deviation Discriminability. As was the case in the condensed table, the alternative hypothesis, indicating significant separation across groups, was accepted for all experiments, with $p < 0.005$.

				**Unscaled Reference**		**Dense Perturbations**		**Sparse Perturbations**	
Exp.	Subj.	Sess.	Samp.	Det.	Prob.	Det.	Prob.	Det.	Prob.
1.1	All	All	1	0.64 ± 0.00	0.65 ± 0.00	0.82 ± 0.00	0.82 ± 0.00	0.77 ± 0.00	0.75 ± 0.00
1.2	All	1	All	1.00 ± 0.00	1.00 ± 0.00	1.00 ± 0.00	1.00 ± 0.00	0.93 ± 0.02	0.90 ± 0.02
1.3	All	1	1			1.00 ± 0.00	1.00 ± 0.00	0.94 ± 0.02	0.90 ± 0.02
2.4	1	All	All	1.00 ± 0.00	1.00 ± 0.00	1.00 ± 0.00	1.00 ± 0.00	0.88 ± 0.12	0.85 ± 0.12
2.5	1	All	1			1.00 ± 0.00	1.00 ± 0.00	0.89 ± 0.11	0.84 ± 0.12
3.6	1	1	All			0.99 ± 0.03	1.00 ± 0.00	0.71 ± 0.07	0.61 ± 0.05

subsamples or simulations, respectively. For this reason, it is unsurprising that the dataset achieved considerably higher discriminability scores.

Session Variation Similar to subject variation, the session variation was also modelled across either both or a single subsample in experiments 2.4 and 2.5. In both of these cases the performance was similar, and the finding that sparse perturbations reduced the off-target signal was consistent.

Scaling of discriminability with N When samples were added to the dataset across perturbed executions, the discriminability statistic inflated to a plateau even when no information was added (e.g. the dataset was replicated). This effect is demonstrated for the reference executions and is shown in Figure 5. As we can see, the reference discriminability scores without data duplication (unscaled) were 0.64 and 0.65 for the deterministic and probabilistic pipelines, respectively. After duplicating the dataset 20 times, matching the size of the 20-sample perturbed dataset, we can see that this (scaled) score plateaus at 0.82 for both pipelines. For consistency, in the main body of the text the reference execution performance was communicated as the scaled quantity.

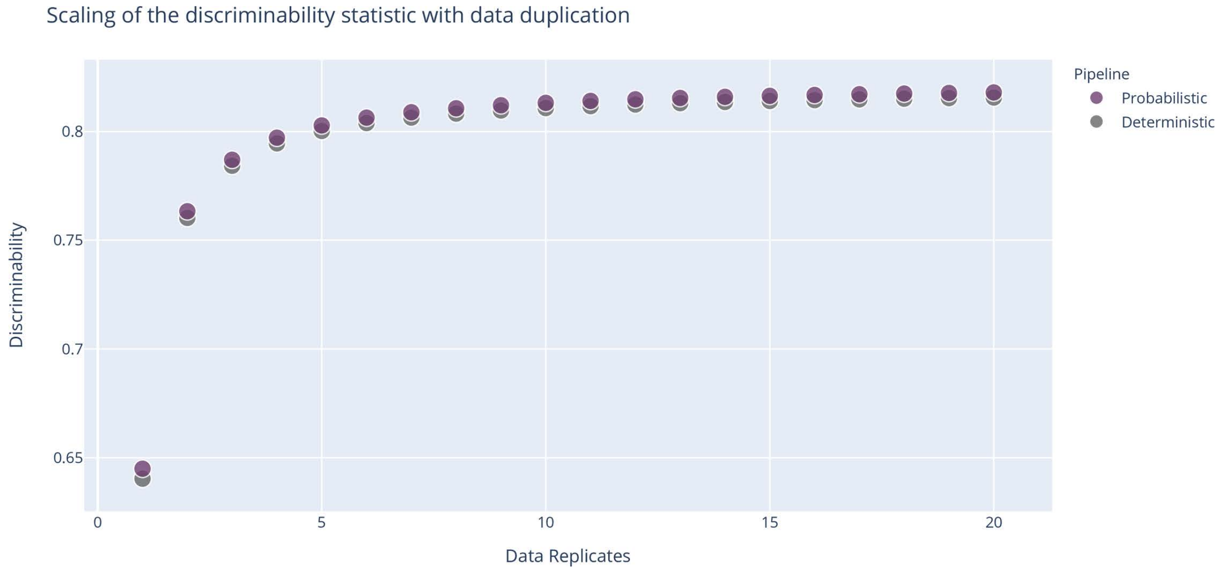

Fig 5. Scaling behaviour of the discriminability statistic with data duplication.

Supplemental Section S3 Univariate Graph Statistics

Figure 6 explores the stability of univariate graph-theoretical metrics computed from the perturbed graphs, including modularity, global efficiency, assortativity, average path length, and edge count. When aggregated across individuals and perturbations, the distributions of these statistics (Figures 6A and 6B) showed no significant differences between perturbation methods for either deterministic or probabilistic pipelines, consistent with the comparison of the cumulative density of the multivariate statistics compared in Fig 2.

However, when quantifying the stability of these measures across connectomes derived from a single session of data, the two perturbation methods show considerable differences. The number of significant digits in univariate statistics for dense perturbation instrumented connectome generation exceeded 11 digits for all measures except modularity, which contained more than 4 significant digits of information (Figure 6C). When detecting false-positives from the distributions of observed statistics for a given session, the rate (using a threshold of $p = 0.05$) was approximately 2% for all statistics with the exception of modularity which again was less stable with an approximately 10% false positive rate. The probabilistic pipeline is significantly more stable than the deterministic pipeline ($p < 0.0001$; exploratory) for all features except modularity. When similarly evaluating these features from connectomes generated in the sparse perturbation setting, no statistic was stable with more than 3 significant digits or a false positive rate lower than nearly 6% (Figure 6D). The deterministic pipeline was more stable than the probabilistic pipeline in this setting ($p < 0.0001$; exploratory).

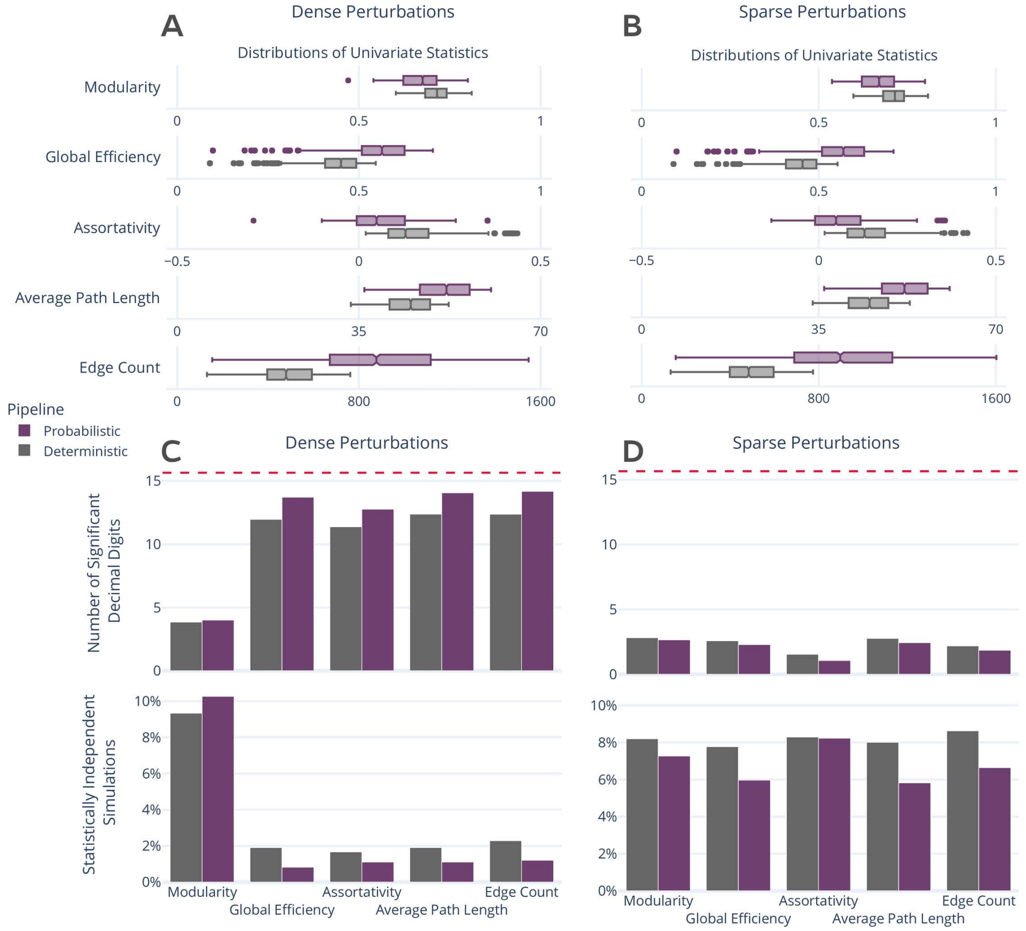

Fig 6. Distribution and stability assessment of univariate graph statistics. (**A**, **B**) The distributions of each computed univariate statistic across all subjects and perturbations for dense and sparse settings, respectively. There was no significant difference between the distributions in A and B. (**C**, **D**; top) The number of significant decimal digits in each statistic across perturbations, averaged across individuals. The dashed red line refers to the maximum possible number of significant digits. (**C**, **D**; bottom) The percentage of connectomes which were deemed significantly different ($p < 0.05$) from the others obtained for an individual.

Two notable differences between the two perturbation methods are, first, the uniformity in the stability of the statistics, and second, the dramatic decline in stability of individual statistics in the sparse perturbation setting despite the consistency in the overall distribution of values. This result is consistent with that obtained from the multivariate exploration performed in the body of this article. It is unclear at present if the discrepancy between the stability of modularity in the pipeline perturbation context versus the other statistics suggests the implementation of this measure is the source of instability or if it is implicit to the measure itself. The dramatic decline in the stability of features derived from sparse perturbed graphs despite no difference in their overall distribution both shows that while individual estimates may be unstable the comparison between aggregates or groups may be considered much more reliable; this finding is consistent with that presented for multivariate statistics.

Reference

1. H. Huang and M. Ding, "Linking functional connectivity and structural connectivity quantitatively: a comparison of methods," *Brain connectivity*, vol. 6, no. 2, pp. 99–108, 2016.
