# Supplementary material for: Numerical uncertainty in analytical pipelines lead to impactful variability in brain networks
Source: PLoS One. 2021 Nov 1;16(11):e0250755. doi: 10.1371/journal.pone.0250755 (PMC8559953; doi:10.1371/journal.pone.0250755)
Supplement: Attachment — Submitted filename: response_to_reviewers_impact_of_instability.pdf [file pone.0250755.s002.pdf]

July 30th, 2021

Dear reviewers and PLOS ONE editorial staff,

Please find below a response letter indicating how we have addressed the feedback provided in the reviews of our manuscript. The reviewers' comments have been indented with responses beneath them. Changes to the text of our article have been marked in blue within the document and, where relevant, excerpts of text have been presented here for clarity.

Thank you,
Gregory Kiar, PhD, on behalf of my coauthors

Reviewer 1

> The present study deals with the notion of reproducibility which makes research difficult to make progress nowadays. The manuscript is well-written but titles like introduction and so on are missing making it difficult to follow.

Thank you for this note. The headings "Introduction" and "Results" have been added, and "Methods" has been updated to "Materials and Methods".

> 1. Abstract
> References should be avoided from the abstract and the abstract should be reword to reveal better the novelty/findings of the present work

Thank you for the feedback, references have been removed from the abstract and the text has been modified accordingly to more explicitly state the findings of the work. The new abstract is as follows:

> The analysis of brain-imaging data requires complex processing pipelines to support findings on brain function or pathologies. Recent work has shown that variability in analytical decisions, small amounts of noise, or computational environments can lead to substantial differences in the results, endangering the trust in conclusions. We explored the instability of results by instrumenting a structural connectome estimation pipeline with Monte Carlo Arithmetic to introduce random noise throughout. We evaluated the reliability of the connectomes, the robustness of their features, and the eventual impact on analysis. The stability of results was found to range from perfectly stable (i.e. all digits of data significant) to highly unstable (i.e. 0 – 1 significant digits). This paper highlights the potential of leveraging induced variance in estimates of brain connectivity to reduce the bias in networks without compromising reliability,

alongside increasing the robustness and potential upper-bound of their applications in the classification of individual differences. We demonstrate that stability evaluations are necessary for understanding error inherent to brain imaging experiments, and how numerical analysis can be applied to typical analytical workflows both in brain imaging and other domains of computational sciences, as the techniques used were data and context agnostic and globally relevant. Overall, while the extreme variability in results due to analytical instabilities could severely hamper our understanding of brain organization, it also affords us the opportunity to increase the robustness of findings.

> 2. Introduction (even though there is not title for this)
> 2nd paragraph: The problem here is not only the variability among toolboxes but also among subjects with regard for example their dielectric properties. See for example Antonakakis et al., 2020, NeuroImage (https://pubmed.ncbi.nlm.nih.gov/32919058/). Inter-subject variability of skull conductivity is a special parameter that can really affect the reproduction of a neuroimaging result.

Thank you for this example, we have added a sentence which expands our point about tool variability to datasets as well, and cited this work. The sentence added to the end of this paragraph is as follows:

The previous study does not broach dataset differences, but there are considerable works which demonstrate that data selection may compound these effects (e.g. 14, 16).

> 3. A strong concern of mine is if there is any kind of False detection rate applied on the statistical comparison throughout the entire study?

Thank you for pointing this out. While this work largely does not report p-values or claim to test hypotheses, the p-values reported had been adjusted for multiple comparisons. The caption for Table 1 and the Evaluation section of the methods were updated to reflect this, now including the phrase "and corrected for multiple comparisons".

> Page 6, last paragraph the classification model is not described well, the author need to name the classifier they used. 'logistic regression classifier is one case and it might be weak for the purpose of this study.

Thank you for this note. While our technique was described in the method, we agree that it is worth stating it explicitly here. We have added a sentence to this paragraph to clarify the details of this method and explicitly recognize that these methods and performance values are comparable to those previously noted in the field. The excerpt is as follows:

In particular, we used Principal Component Analysis followed by a Logistic Regression classifier to predict BMI label, and demonstrated similar performance to previous work which adopted similar techniques for this task (31, 32). We compared the performance achieved across numerically perturbed samples to both the reference and random performance (Figure 3).

Academic Editor

1) Your analysis cannot be generalized to all the different types of brain connectomes, unless you extended further the description of your analysis.

If this editor wishes to provide specific feedback which they believe are lacking within our paper we would be happy to update the manuscript, accordingly. Until such time, we believe that the Methods section (which, based on the following feedback, the editor may have missed) describes our data, experiment, and analysis techniques in sufficient detail for replication and adoption and would welcome concrete feedback to the contrary.

There is no title in the introduction, no material & methods, and no results section.

Thank you for the note. Titles have been added to the introduction and results sections, which were not labeled. The methods section had already been labeled as such.

2) You analyzed dMRI and explore how numerical errors can violate brain network topologies in terms of brain fingerprinting, repeatability across sessions, and brain-phenotype relationships.There is no description of the dataset. You can add it in supp. material

The dataset was described in the first subsection of the Methods section, including a citation to the original paper which describes the collection in detail. This section of ours clearly stated the selected sample, their demographic data, and specific details about the data acquisition.

3) It's difficult to understand the whole idea behind your study and types of perturbations (sparse and dense) etc. You didn't compare for example two independent software that implement the same tractographic algorithm in order to explore their outcome.

Thank you for this comment, we have added a clarification to the subsection of the Discussion with regards to shortcomings to explicitly address this point. It is as follows:

Similarly, this complexity along with the added layer of difficulty in comparing instrumentations meant that only algorithms within a single library were tested.

> The whole study demands substantial revision and rewriting explaining further the rationale and the source of numerical uncertainties.

We have addressed the comments mentioned previously by the reviewer and editor.

> Also, why did you generalize the outcome of your experiments across every type of modality-based brain networks?

Throughout our manuscript we did not claim that our specific results would apply across "every type of modality-based brain networks," but that a) the techniques could and should be applied broadly and that b) diffusion MRI-based networks were unlikely to be more susceptible to these issues than any other modality due to the fundamental cause of numerical imprecision. We have made it more explicit that we are referring to structural connectomes at various points throughout the text.

Journal Requirements

We have adjusted our acknowledgements section to no longer include funding information. The funding statement previously provided should not be changed.
