# Supplementary material for: Numerical uncertainty in analytical pipelines lead to impactful variability in brain networks
Source: PLoS One. 2021 Nov 1;16(11):e0250755. doi: 10.1371/journal.pone.0250755 (PMC8559953; doi:10.1371/journal.pone.0250755)
Supplement: Peer review history

# Peer review: Decision Letter 0

**DOI:** 10.1371/journal.pone.0250755.r001

**Authors:** Stavros I. Dimitriadis

30 Jul 2021

PONE-D-21-12648

Numerical Uncertainty in Analytical Pipelines Lead to Impactful Variability in Brain Networks

PLOS ONE

Dear Dr. Kiar,

Thank you for submitting your manuscript to PLOS ONE. After careful consideration, we feel that it has merit but does not fully meet PLOS ONE’s publication criteria as it currently stands. Therefore, we invite you to submit a revised version of the manuscript that addresses the points raised during the review process.

==============================

The authors explored how Numerical Uncertainty in Analytical Pipelines can alter brain network topology.

The study is interesting but further clarifications and modifications are needed.

Below, I summarize my comments:

1) Your analysis cannot be generalized to all the different types of brain connectomes, unless you extended further the description of your analysis.

    There is no title in the introduction, no material & methods, and no results section.

   

2) You analyzed dMRI and explore how numerical errors can violate brain network topologies in terms of

    brain fingerprinting, repeatability across sessions, and brain-phenotype relationships.

    There is no description of the dataset. You can add it in supp. material

3) It's difficult to understand the whole idea behind your study and types of perturbations (sparse and dense) etc.

    You didn't compare for example two independent software that implement the same tractographic algorithm in order

    to explore their outcome.

    The whole study demands substantial revision and rewriting explaining further the rationale and the source of numerical uncertainties.

    Also, why did you generalize the outcome of your experiments across every type of modality-based brain networks?    

==============================

Please submit your revised manuscript by Sep 13 2021 11:59PM. If you will need more time than this to complete your revisions, please reply to this message or contact the journal office at plosone@plos.org. When you're ready to submit your revision, log on to https://www.editorialmanager.com/pone/ and select the 'Submissions Needing Revision' folder to locate your manuscript file.

Please include the following items when submitting your revised manuscript:

- A rebuttal letter that responds to each point raised by the academic editor and reviewer(s). You should upload this letter as a separate file labeled 'Response to Reviewers'.
- A marked-up copy of your manuscript that highlights changes made to the original version. You should upload this as a separate file labeled 'Revised Manuscript with Track Changes'.
- An unmarked version of your revised paper without tracked changes. You should upload this as a separate file labeled 'Manuscript'.

If you would like to make changes to your financial disclosure, please include your updated statement in your cover letter. Guidelines for resubmitting your figure files are available below the reviewer comments at the end of this letter.

If applicable, we recommend that you deposit your laboratory protocols in protocols.io to enhance the reproducibility of your results. Protocols.io assigns your protocol its own identifier (DOI) so that it can be cited independently in the future. For instructions see: http://journals.plos.org/plosone/s/submission-guidelines#loc-laboratory-protocols. Additionally, PLOS ONE offers an option for publishing peer-reviewed Lab Protocol articles, which describe protocols hosted on protocols.io. Read more information on sharing protocols at https://plos.org/protocols?utm_medium=editorial-email&utm_source=authorletters&utm_campaign=protocols.

We look forward to receiving your revised manuscript.

Kind regards,

Stavros I. Dimitriadis

Academic Editor

PLOS ONE

Journal Requirements:

When submitting your revision, we need you to address these additional requirements.

1. Please ensure that your manuscript meets PLOS ONE's style requirements, including those for file naming. The PLOS ONE style templates can be found at

https://journals.plos.org/plosone/s/file?id=wjVg/PLOSOne_formatting_sample_main_body.pdf and

https://journals.plos.org/plosone/s/file?id=ba62/PLOSOne_formatting_sample_title_authors_affiliations.pdf

2. Thank you for stating the following in the Acknowledgments Section of your manuscript:

”This research was financially supported by the Natural Sciences and Engineering Research Council of Canada (NSERC) (award 360 no. CGSD3-519497-2018). This work was also supported in part by funding provided by Brain Canada, in partnership with 361 Health Canada, for the Canadian Open Neuroscience Platform initiative. “

We note that you have provided additional information within the Acknowledgements Section that is not currently declared in your Funding Statement. Please note that funding information should not appear in the Acknowledgments section or other areas of your manuscript. We will only publish funding information present in the Funding Statement section of the online submission form.

Please remove any funding-related text from the manuscript and let us know how you would like to update your Funding Statement. Currently, your Funding Statement reads as follows:

”GK was fully supported for this work by the Natural Sciences and Engineering Research Council of Canada <https://www.nserc-crsng.gc.ca/index_eng.asp> Award number: CGSD3 - 519497 - 2018. The funders had no role in study design, data collection and analysis, decision to publish, or preparation of the manuscript.”

Please include your amended statements within your cover letter; we will change the online submission form on your behalf.

Additional Editor Comments (if provided):

The authors explored how Numerical Uncertainty in Analytical Pipelines can alter brain network topology.

The study is interesting but further clarifications and modifications are needed.

Below, I summarize my comments:

1) Your analysis cannot be generalized to all the different types of brain connectomes, unless you extended further the description of your analysis.

There is no title in the introduction, no material & methods, and no results section.

2) You analyzed dMRI and explore how numerical errors can violate brain network topologies in terms of

brain fingerprinting, repeatability across sessions, and brain-phenotype relationships.

There is no description of the dataset. You can add it in supp. material

3) It's difficult to understand the whole idea behind your study and types of perturbations (sparse and dense) etc.

You didn't compare for example two independent software that implement the same tractographic algorithm in order

to explore their outcome.

The whole study demands substantial revision and rewriting explaining further the rationale and the source of numerical uncertainties.

Also, why did you generalize the outcome of your experiments across every type of modality-based brain networks?

[Note: HTML markup is below. Please do not edit.]

Reviewers' comments:

Reviewer's Responses to Questions

**Comments to the Author**

1. Is the manuscript technically sound, and do the data support the conclusions?

The manuscript must describe a technically sound piece of scientific research with data that supports the conclusions. Experiments must have been conducted rigorously, with appropriate controls, replication, and sample sizes. The conclusions must be drawn appropriately based on the data presented. 

Reviewer #1: Yes

**********

2. Has the statistical analysis been performed appropriately and rigorously? 

Reviewer #1: No

**********

3. Have the authors made all data underlying the findings in their manuscript fully available?

The PLOS Data policy requires authors to make all data underlying the findings described in their manuscript fully available without restriction, with rare exception (please refer to the Data Availability Statement in the manuscript PDF file). The data should be provided as part of the manuscript or its supporting information, or deposited to a public repository. For example, in addition to summary statistics, the data points behind means, medians and variance measures should be available. If there are restrictions on publicly sharing data—e.g. participant privacy or use of data from a third party—those must be specified.

Reviewer #1: Yes

**********

4. Is the manuscript presented in an intelligible fashion and written in standard English?

PLOS ONE does not copyedit accepted manuscripts, so the language in submitted articles must be clear, correct, and unambiguous. Any typographical or grammatical errors should be corrected at revision, so please note any specific errors here.

Reviewer #1: Yes

**********

5. Review Comments to the Author

Please use the space provided to explain your answers to the questions above. You may also include additional comments for the author, including concerns about dual publication, research ethics, or publication ethics. (Please upload your review as an attachment if it exceeds 20,000 characters)

Reviewer #1: The present study deals with the notion of reproducibility which makes research difficult to make progress nowadays. The manuscript is well-written but titles like introduction and so on are missing making it difficult to follow.

A few comments before the acceptance of this work

1. Abstract

References should be avoided from the abstract and the abstract should be reword to reveal better the novelty/findings of the present work

2. Introduction (even though there is not title for this)

2nd paragraph: The problem here is not only the variability among toolboxes but also among subjects with regard for example their dielectric properties. See for example Antonakakis et al., 2020, NeuroImage (https://pubmed.ncbi.nlm.nih.gov/32919058/). Inter-subject variability of skull conductivity is a special parameter that can really affect the reproduction of a neuroimaging result.

3. A strong concern of mine is if there is any kind of False detection rate applied on the statistical comparison throughout the entire study?

Page 6, last paragraph the classification model is not described well, the author need to name the classifier they used. 'logistic regression classifier is one case and it might be weak for the purpose of this study.

**********

6. PLOS authors have the option to publish the peer review history of their article (what does this mean?). If published, this will include your full peer review and any attached files.

If you choose “no”, your identity will remain anonymous but your review may still be made public.

**Do you want your identity to be public for this peer review?** For information about this choice, including consent withdrawal, please see our Privacy Policy.

Reviewer #1: No

[NOTE: If reviewer comments were submitted as an attachment file, they will be attached to this email and accessible via the submission site. Please log into your account, locate the manuscript record, and check for the action link "View Attachments". If this link does not appear, there are no attachment files.]

While revising your submission, please upload your figure files to the Preflight Analysis and Conversion Engine (PACE) digital diagnostic tool, https://pacev2.apexcovantage.com/. PACE helps ensure that figures meet PLOS requirements. To use PACE, you must first register as a user. Registration is free. Then, login and navigate to the UPLOAD tab, where you will find detailed instructions on how to use the tool. If you encounter any issues or have any questions when using PACE, please email PLOS at figures@plos.org. Please note that Supporting Information files do not need this step.

# Author comment: Author response to Decision Letter 0

**DOI:** 10.1371/journal.pone.0250755.r002

4 Aug 2021

The response to reviewers was provided in a supplied attachment.

Attachment Submitted filename: response_to_reviewers_impact_of_instability.pdf Click here for additional data file.

# Editor report: Decision Letter 1

**DOI:** 10.1371/journal.pone.0250755.r003

**Authors:** Stavros I. Dimitriadis

26 Aug 2021

Numerical Uncertainty in Analytical Pipelines Lead to Impactful Variability in Brain Networks

PONE-D-21-12648R1

Dear Dr. Kiar,

We’re pleased to inform you that your manuscript has been judged scientifically suitable for publication and will be formally accepted for publication once it meets all outstanding technical requirements.

Within one week, you’ll receive an e-mail detailing the required amendments. When these have been addressed, you’ll receive a formal acceptance letter and your manuscript will be scheduled for publication.

An invoice for payment will follow shortly after the formal acceptance. To ensure an efficient process, please log into Editorial Manager at http://www.editorialmanager.com/pone/, click the 'Update My Information' link at the top of the page, and double check that your user information is up-to-date. If you have any billing related questions, please contact our Author Billing department directly at authorbilling@plos.org.

If your institution or institutions have a press office, please notify them about your upcoming paper to help maximize its impact. If they’ll be preparing press materials, please inform our press team as soon as possible -- no later than 48 hours after receiving the formal acceptance. Your manuscript will remain under strict press embargo until 2 pm Eastern Time on the date of publication. For more information, please contact onepress@plos.org.

Kind regards,

Stavros I. Dimitriadis

Academic Editor

PLOS ONE

Additional Editor Comments (optional):

I have no further comments.

Reviewers' comments:

# Editor report: Acceptance letter

**DOI:** 10.1371/journal.pone.0250755.r004

**Authors:** Stavros I. Dimitriadis

12 Oct 2021

PONE-D-21-12648R1 

Numerical Uncertainty in Analytical Pipelines Lead to Impactful Variability in Brain Networks 

Dear Dr. Kiar:

I'm pleased to inform you that your manuscript has been deemed suitable for publication in PLOS ONE. Congratulations! Your manuscript is now with our production department. 

If your institution or institutions have a press office, please let them know about your upcoming paper now to help maximize its impact. If they'll be preparing press materials, please inform our press team within the next 48 hours. Your manuscript will remain under strict press embargo until 2 pm Eastern Time on the date of publication. For more information please contact onepress@plos.org.

If we can help with anything else, please email us at plosone@plos.org. 

Thank you for submitting your work to PLOS ONE and supporting open access. 

Kind regards, 

PLOS ONE Editorial Office Staff

on behalf of

Dr. Stavros I. Dimitriadis 

Academic Editor

PLOS ONE
